# Reporting guideline checklists are not quality evaluation forms: they are guidance for writing

**DOI:** 10.1002/hsr2.165

**Published:** 2020-05-03

**Authors:** Patricia Logullo, Angela MacCarthy, Shona Kirtley, Gary S. Collins

**Affiliations:** ^1^ UK EQUATOR Centre, Centre for Statistics in Medicine, Nuffield Department of Orthopaedics & Musculoskeletal Sciences University of Oxford Oxford UK; ^2^ NIHR Oxford Biomedical Research Centre John Radcliffe Hospital Oxford UK

**Keywords:** 4‐6, MesH

One of the fundamental principles of health research integrity is that research methods and results should be completely and transparently reported. Clear, detailed reporting allows the reader to understand how a study was designed and conducted, to judge the reliability of its findings and the reproducibility of its methods, and to use the tested interventions in their clinical practice.^1^, ^2^, ^3^ The way in which research results are reported, therefore, can have a direct impact on patients' lives.^4^ As the late Professor Douglas Altman said, ‘*Readers should not have to infer what was probably done, they should be told explicitly’*.^5^

Reporting guidelines were created to help researchers write reports that contain the minimum set of information necessary to allow readers to clearly understand what was done and found in a study and facilitate a formal risk of bias assessment (using tools such as the Cochrane Risk of Bias tool or QUADAS). Complete reporting can also allow replication of study methods and procedures. A reporting guideline is ‘*a checklist, flow diagram, or explicit text to guide authors in reporting a specific type of research, developed using explicit methodology’*.^6^ Following the publication of the first reporting guideline for clinical trials, CONSORT, in 1996,^7^ multiple reporting guidelines have been published, covering a range of study designs (eg, clinical trials, observational studies), clinical areas (eg, nutrition), or parts of a report (eg, abstracts), to help biomedical researchers write up their studies for publication.^8^, ^9^ Stakeholders in biomedical research have embraced reporting guidelines, with major funders and a large number of biomedical journals endorsing the guidelines and increasingly requiring their use.^10^, ^11^

The most widely used and well‐known reporting guidelines usually consist of a statement paper that describes the process of developing the guideline and presents the guideline usually in the form of a ‘checklist’.^4^ Each checklist consists of a different number of reporting content items, ranging from just a few to more than 30 items. These checklists are designed to be easy to use by authors when they start writing their manuscript. Many journals have recognised how useful they are and have implemented reporting guidelines in their submission and editorial processes. Several journals also require authors to submit a completed checklist indicating where in the manuscript each item has been reported.

Reporting guidelines are (or at least should be) rigorously developed following an extensive process of expert consultation and should not reflect just the opinion of one individual^6^; they should represent a consensus‐based minimal set of items that a group of experienced researchers, journal editors, policymakers, and other stakeholders (eg, funders, patient representatives) have determined should be reported.

## WHAT IS THE OUTCOME BEING MEASURED?

Whilst designed to help improve the completeness and transparency of reporting, reporting guidelines are increasingly used to determine the ‘quality’ of a research paper. However, there are many problems with this. One major issue relates to the concept of quality itself. While some researchers might think that a 100% adherence to a set of content reporting items would mean ‘a quality paper’, others might argue that this ‘top quality’ is not attainable and manuscripts adhering to, say, 80% of the items are ‘well reported’. Therefore, there should first be a consensus—ideally agreed by reporting guideline authors—about determining what level of quality is needed for a health research article to be considered ‘well reported’; in other words, define what quality of reporting is. This is, however, what properly developed reporting guidelines do: they outline a minimum set of information that should be reported in health research manuscripts. This minimum set of information items compose and define a ‘total quality’ report, and researchers should ensure that they indeed describe every item in their manuscripts.

However, if one defines ‘reporting quality’ as 100% adherence to a reporting checklist, understood as the adherence to all items of a given reporting guideline, then it will be virtually impossible to find a ‘good report’ in currently published research. On the other hand, if the outcome is too broadly defined and not standardized, such flexibility might put two very different papers under the same category of ‘good report’. For example, the same manuscript may be evaluated as a ‘good report’ by a study considering 70% of adherence to a reporting guideline, while another study would find this same manuscript not so good because the authors expected 80% to be a minimum adherence indicating quality. Similarly, manuscripts may have the same level of adherence but cover different aspects of the reporting guideline, as different researchers can consider different items as key or ancillary. ‘Reporting quality’, therefore, is a very subjective concept. Published studies do not agree on how much quality to expect—and maybe they should all expect 100% adherence as per the definition of reporting guidelines: a **minimum** set of information.

## QUALITY EVALUATION TOOLS?

Numerous studies have now been published evaluating whether individual reporting guidelines have made any improvement to the completeness of published reports.^12^, ^13^, ^14^ These studies typically use adherence to a reporting guideline as a surrogate for reporting quality^15^, ^16^, ^17^, ^18^, ^19^, ^20^, ^21^, ^22^, ^23^, ^24^, ^25^, ^26^, ^27^, ^28^, ^29^, ^30^, ^31^, ^32^, ^33^, ^34^, ^35^, ^36^, ^37^, ^38^, ^39^, ^40^, ^41^ or even, inadequately, for study quality.^42^ The findings of such research‐on‐research studies generally agree that the quality of health research reports is still lacking.^43^ However, the methods used to investigate this complex concept of ‘quality of publication’ varies widely in the literature. In most cases, the original reporting guideline checklist is being used without modification to measure ‘quality’—which is a complex concept on its own—but there is no consensus on whether or how to apply these reporting guidelines in studies on adherence.

One might argue that because reporting guidelines are the result of carefully planned discussions at consensus meetings, their face validity would be guaranteed, in the sense that all items in the checklist are considered relevant or essential. However, that does not mean that when experts develop reporting checklists, they do so with the intention that the checklist will also serve as a properly designed evaluation tool for assessing reporting quality; reporting guidelines are specifically designed as guidance for writing. The STREGA reporting guideline explicitly indicates this: ‘*the STREGA reporting guidelines should not be used for screening submitted manuscripts to determine the quality or validity of the study being reported’*.^44^

One exception in the literature, however, is the TRIPOD guideline.^45^, ^46^, ^47^ The TRIPOD Statement is a reporting guideline for prediction models (TRIPOD stands for *Tr*ansparent *R*eporting of a multivariable prediction model for *I*ndividual *P*rognosis *O*r *D*iagnosis).^45^, ^46^, ^47^ TRIPOD authors, recognising the widespread secondary use of reporting guidelines, set out to develop and publish an evaluation form for assessing the quality of reporting of diagnostic and prognostic prediction model studies. This form can be used by any researcher trying to evaluate the quality of prediction models in the literature, facilitating the comparison of results of different studies (Table 1).^47^, ^48^

**TABLE 1:** Example of checklist items turned into evaluation form questions in the TRIPOD reporting guideline, for prediction models for prognosis or diagnosis

Item	Original reporting guideline checklist item	Evaluation form items					
		#	Evaluation form question	Instructions for scoring			
				D Score 1 if element is scored as ‘Y’	V Score 1 if element is scored as ‘Y’	IV Score 1 if element is scored as ‘Y’	D + V Score 1 if element is scored as ‘Y’
4a	‘Describe the study design or source of data (eg, randomized trial, cohort, or registry data), separately for the development and validation data sets, if applicable’.	i	The study design/source of data is described	Y/N	Y/N	Y/N	=Y if D4ai = Y AND V4ai = Y
			For example, Prospectively designed, existing cohort, existing RCT, registry/medical records, case control, case series.				
			This needs to be explicitly reported; reference to this information in another article alone is insufficient.				

Item	Original reporting guideline checklist item	Evaluation form items					
		#	Evaluation form question	Instructions for scoring			
				D Score 1 if all elements are scored as ‘Y’, ‘NA’, or ‘R’	V Score 1 if all elements are scored as ‘Y’, ‘NA’, or ‘R’	IV Score 1 if all elements are scored as ‘Y’, ‘NA’, or ‘R’	D + V Score 1 if all elements are scored as ‘Y’, ‘NA’, or ‘R’
4b	‘Specify the key study dates, including start of accrual; end of accrual; and, if applicable, end of follow‐up’.	i	The starting date of accrual is reported	Y/N/R	Y/N/R	Y/N/R	=Y if (D4bi = Y AND V4bi = [Y OR R]) OR (D4bi = [Y OR R] AND V4bi = Y) = R if D4bi = R AND V4bi = R
		ii	The end date of accrual is reported	Y/N/R	Y/N/R	Y/N/R	=Y if (D4bii = Y AND V4bii = [Y OR R]) OR (D4bii = [Y OR R] AND V4bii = Y) = R if D4bii = R AND V4bii = R
		iii	The length of follow‐up and prediction horizon/time frame are reported, if applicable	Y/N/NA	Y/N/NA	Y/N/NA	=Y if (D4biii = Y AND V4biii = [Y OR NA]) OR (D4biii = [Y OR NA] AND V4biii = Y) = NA if D4biii = NA AND V4biii = NA
			E.g. ‘Patients were followed from baseline for 10 years’ and ‘10‐year prediction of…’; notably for prognostic studies with long term follow‐up.				
			If this is not applicable for an article (ie, diagnostic study or no follow‐up), then score Not applicable.				

Abbreviations: Y, yes; N, no; N/A, not applicable; R, referenced; D, development (applies for studies that develop new prediction models); V, external validation (applies for studies that validate existing models); IV, applies for studies of incremental value; D + V, applies for studies of development and external validation of the same model.

Table 1 shows an example of one checklist item (item 4) from the TRIPOD reporting guideline. The exact text from the TRIPOD reporting checklist is contained in column 1. Column 2 provides the text from the TRIPOD evaluation tool, which breaks down the item into several questions. Columns 3 to 6 provide information about how to score the reporting of item 4. The Table shows that in order to conduct a robust evaluation of the reporting of checklist items, simply relying on the reporting checklist items themselves is not enough. Each item needs to be broken down into appropriate questions, with an accompanying scoring system developed. Building such an evaluation tool for each reporting guideline will enable researchers to consistently scrutinise and score the reporting quality of research papers, with every researcher around the world using the same tool, as it happens with quality of life evaluations, for example, an outcome that can be compared among studies when they use the same tool.^49^, ^50^

## SCORING SYSTEMS

Another important issue is the design and content of the data extraction form used to evaluate ‘reporting quality’ in these studies. How do researchers assign a score to each reporting checklist item in these evaluation forms? Currently, there seems to be no consistency in the methods or scoring systems being used by researchers.^15^, ^16^, ^17^, ^18^, ^19^, ^20^, ^21^, ^22^, ^23^, ^24^, ^25^, ^26^, ^27^, ^28^, ^29^, ^30^, ^31^, ^32^, ^33^, ^34^, ^35^, ^36^, ^37^, ^38^, ^39^, ^40^ Some studies evaluate simply whether an item is reported or not (a ‘yes/no’ dichotomised score).^19^, ^25^, ^29^ Others assign three options, for example, ‘not reported’, ‘fully reported’, and ‘partially reported’ or ‘not applicable’.^15^, ^17^, ^20^, ^21^, ^22^, ^23^, ^24^, ^26^, ^27^, ^31^, ^33^, ^37^, ^38^, ^39^, ^40^ Some studies also use more options, such as a five‐point scale of quality for each item.^28^, ^32^, ^35^ Given the variability in scoring adherence between studies (ie, each study gives different weights to the same item), how can the results of these studies be compared?

One might propose that it is sufficient to include a ‘not applicable’ option to the reporting guideline checklist items when developing a scoring system, and it would be ready to use as an evaluation tool. But this may not be enough. The authors of TRIPOD discuss:

Overall adherence, in the form of a percentage of items adhered to, requires a clear denominator of total number of items one can adhere to. One has to decide whether to take items that are considered not applicable into account in the numerator as well as in the denominator. Determining applicability is subjective and requires interpretation. In our experience, items for which interpretation was needed, sometimes indicated by phrases like ‘if relevant’ or ‘if applicable,’ were the most difficult ones to score and these items are a potential threat to inter‐assessor agreement.

As the number of papers assessing the quality of reporting of studies is increasing, it is important to highlight the pitfalls of using reporting guideline checklists as evaluation tools. It seems that the only way to prevent multiple methodologists from assessing manuscript quality using different criteria, forms, scoring systems, outcomes, and number of evaluators is to provide clear guidance on how to evaluate the reporting quality of manuscripts and to encourage all reporting guideline developers to publish a reporting evaluation tool together with or soon after the publication of a new reporting guideline. Providing an evaluation form would, at least, offer evaluators a single tool to be used uniformly across studies, allowing some comparability.

## DEVELOPMENT AND TESTING OF EVALUATION TOOLS

There are several methodological steps that researchers must follow when developing evaluation tools to ensure the relevance and robustness of a new tool to evaluate a subjective concept, for instance, quality of life. An evaluation instrument such as a questionnaire or scoring system (ie, composed of multiple parts or items, taken as indirect indicators) must undergo validity testing before it can be said to accurately measure what it intends to measure, that it is clear and easily understandable for users, and that it represents all facets of a (sometimes complex) concept. Where other instruments exist, it is possible to validate the results of a new tool by comparing it to the other, considered, so far, a ‘gold standard’. It is desirable that the instrument has some consistency over time too, measuring the same thing the same way twice, or by different evaluators.

As far as we know, none of these methods traditionally used in health outcome measurement have been followed when developing reporting guideline checklists. Perhaps this is because reporting quality is seen as an objective outcome: the 100% adherence to a checklist. Perhaps it is because the developers did not set out to develop an evaluation tool in the first place, but only guidance for writing, the exception being the TRIPOD evaluation tool, mentioned earlier, which was developed in addition to the reporting guideline checklist.

There are currently at least 84 reporting guidelines under construction, according to the EQUATOR Network registry (https://www.equator-network.org/library/reporting-guidelines-under-development/); more, if we consider that not every development team registers their guideline under development. Developers should consider building evaluation tools along with their reporting guideline. However, when this is not possible (eg, due to lack of funding), they should follow the example of the STREGA authors^51^ and warn researchers not to use their reporting guideline as a quality evaluation tool. Existing reporting guideline groups should also be encouraged to develop evaluation tools for their guidelines. This will ensure that, in the future, all research studies assessing adherence to reporting guidelines or measuring the ‘quality’ of reporting will use robustly and appropriately developed evaluation tools, and the results will be more meaningful and reliable.

## AUTHOR CONTRIBUTIONS

Conceptualization: Patricia Logullo, Gary S. Collins

Data Curation: Patricia Logullo, Angela MacCarthy, Gary S. Collins

Formal Analysis: Patricia Logullo, Gary S. Collins

Funding Acquisition: Gary S. Collins

Resources: Gary S. Collins

Writing ‐ Original Draft: Patricia Logullo, Shona Kirtley, Gary S. Collins

Writing ‐ Review & Editing: Angela MacCarthy, Shona Kirtley, Gary S. Collins

All authors have read and approved the final version of the manuscript.

## CONFLICT OF INTEREST

Gary Collins is involved in the TRIPOD Statement.
